# T_H_17 Cell and Epithelial Cell Crosstalk during Inflammatory Bowel Disease and Carcinogenesis

**DOI:** 10.3389/fimmu.2017.01373

**Published:** 2017-10-25

**Authors:** Jan Kempski, Leonie Brockmann, Nicola Gagliani, Samuel Huber

**Affiliations:** ^1^Department of Medicine, University Medical Center Hamburg-Eppendorf, Hamburg, Germany; ^2^Department of General, Visceral and Thoracic Surgery, University Medical Center Hamburg-Eppendorf, Hamburg, Germany; ^3^Department of Medicine Solna (MedS), Karolinska Institute, Stockholm, Sweden

**Keywords:** Th17 cells, inflammatory bowel diseases, colorectal cancer, enterocytes, cytokines

## Abstract

The intestine is colonized by hundreds of different species of commensal bacteria, viruses, and fungi. Therefore, the intestinal immune system is constantly being challenged by foreign antigens. The immune system, the commensal microbiota, and the intestinal epithelial surface have to maintain a tight balance to guarantee defense against potential pathogens and to prevent chronic inflammatory conditions at the same time. Failure of these mechanisms can lead to a vicious cycle in which a perpetual tissue damage/repair process results in a pathological reorganization of the normal mucosal surface. This dysregulation of the intestine is considered to be one of the underlying causes for both inflammatory bowel disease (IBD) and colorectal cancer. T_H_17 cells have been associated with immune-mediated diseases, such as IBD, since their discovery in 2005. Upon mucosal damage, these cells are induced by a combination of different cytokines, such as IL-6, TGF-β, and IL-1β. T_H_17 cells are crucial players in the defense against extracellular pathogens and have various mechanisms to fulfill their function. They can activate and attract phagocytic cells. Additionally, T_H_17 cells can induce the release of anti-microbial peptides from non-immune cells, such as epithelial cells. The flip side of the coin is the strong potential of T_H_17 cells to be pro-inflammatory and promote pathogenicity. T_H_17 cells have been linked to both mucosal regeneration and inflammation. In turn, these cells and their cytokines emerged as potential therapeutic targets both for inflammatory diseases and cancer. This review will summarize the current knowledge regarding the T_H_17 cell-enterocyte crosstalk and give an overview of its clinical implications.

## Inflammatory Bowel Disease (IBD) and Carcinogenesis

The gastrointestinal tract is essential for the absorption of nutrition and serves as a crucial barrier to protect the host against pathogens. It is colonized by up to 3.8 × 10^13^ microorganisms such as bacteria and fungi (1). The immune system of the intestine and commensal bacteria maintain a delicate and well-regulated homeostasis. However, when this balancing act is disrupted, chronic inflammatory conditions, such as IBD, can occur. The most common manifestations of IBD are Crohn’s disease (CD) and ulcerative colitis (UC). Their symptoms share common hallmarks such as diarrhea, abdominal pain, and relapsing inflammation in the intestine (2). The inflammation in UC patients is limited mostly to the colon with continuous inflammation of the mucosa and submucosa. CD patients suffer from patchy inflammation that causes deep ulcerations and can affect the whole gastrointestinal tract (3).

Even though the prevalence of IBD especially in western countries such as the USA is high (1.3%) and the resulting costs to the health systems are increasing (4), the underlying cause of IBD is still unknown. Twin studies revealed that genetic predispositions to develop IBD exist; however, the concordance rate for IBD in monozygotic twins does not exceed 50%, highlighting the importance of environmental factors, referred to as Exposome, for disease development (5–7). Furthermore, the intestinal microbiome in combination with the immune system seems to play a crucial role in IBD. Several studies describe a reduced diversity of commensal bacteria species in patients suffering from IBD (8, 9). Additionally, several bacterial pathogens have been implicated in the onset or progression of the disease (10). Furthermore, mouse studies indicate that changes in the microbiota composition can occur prior to colitis development (11, 12), suggesting that microbial changes might be involved in the development of IBD. However, whether dysbiosis is a cause or a consequence of IBD in humans remains to be solved. Nevertheless, barrier defects which are typically present in IBD seem to cause a dysregulated immune response against so far unknown components of the commensals. Especially the adaptive immunity, more specifically CD4^+^ T cells, seems to be inappropriately activated in response to commensal microorganisms in IBD. Classically, CD used to be associated with a chronic T_H_1 immunity, whereas during UC T_H_2 immunity has been thought to be implicated (13). However, after the discovery of the involvement of IL-23 and T_H_17 cells in autoimmune inflammation of the nervous system, further mouse studies revealed a prominent involvement of these cells during intestinal inflammation in CD and UC (14–16). Furthermore, already in 2003, T_H_17 cell-associated cytokines were reported to be upregulated in tissue biopsies and serum of patients with IBD (17, 18). Moreover, chronic inflammation predisposes IBD patients to the development of colorectal cancer (CRC) (19). The chronic inflammation and mucosal injury can trigger long-lasting healing responses that are not terminated, leading to tissue dysfunction and finally to carcinogenesis (20). T_H_17 cells and T_H_17 cell-associated cytokines are also involved during CRC in humans. T_H_17 cells were found elevated in tumors of CRC patients and an increased T_H_17 cell immune response correlates with advanced stages of CRC (21–23).

In summary, four main components lead to the pathology of IBD: genetic predisposition, environmental factors, intestinal microbiome, and a dysregulated immune system. So far, the main therapeutics broadly available to treat IBD are based on suppressing the immune response. But these therapies are unable to reset the intestinal homeostasis and do not directly treat the underlying cause of the chronic inflammation. Therefore, patients suffering from IBD mostly require lifelong treatment. T_H_17 cells could be the link between these four components. Thus, a better understanding of the interactions of these four components and T_H_17 cells is a main focus of current research, with the aim of developing more specific and efficient therapies. This review aims to summarize the current knowledge about the interactions of T_H_17 cells and T_H_17 cell-associated cytokines with the mucosal surface in the intestine and in the microbiota.

## T_H_17 Cells and Their Associated Cytokines

T_H_17 cell-mediated immunity is essential for the clearance of extracellular bacteria and fungi by attracting neutrophils and inducing the release of anti-microbial peptides from epithelial cells (24–28). T_H_17 cell cytokines include IL-17A, IL-17F, TNF-α, and IL-22 (15, 26). It has been demonstrated in mice that intestinal T_H_17 cells are induced by segmented filamentous bacteria (SFB), which are gram-positive, spore-forming bacteria located in the terminal ileum of the small intestine. Accordingly, T_H_17 cells are mainly located in this part of the intestine under physiological conditions (29). A critical feature of SFB is its ability to adhere to the intestinal epithelial cells (IEC). SFB and other bacteria with the same ability such as *Citrobacter rodentium* and *Escherichia coli* induce the production of serum amyloid A (SAA) from epithelial cells (30). Subsequently, SAA induces the release of IL-6, TGF-β, and IL-1β from intestinal cells, especially dendritic cells, which leads to the differentiation of T_H_17 cells (29–31). IL-6 signaling leads to the activation of STAT3 and subsequent induction of RORγt, one of the key transcription factors of T_H_17 cells, and of other T_H_17 cell-related factors such as IL-17A/F and IL-23R (25, 32–34). IL-1β is crucial for the differentiation of T_H_17 cells (35). Besides other effects, IL-1β induces the expression of the transcription factor IRF4, which is needed for the expression of RORγt (36). The role of TGF-β for T_H_17 cell differentiation is still controversial. T_H_17 cells can occur in the absence of TGF-β in the gut mucosa (37). However, TGF-β can negatively regulate T_H_1 and T_H_2 while promoting T_H_17 cell differentiation and therefore favors the contribution of T_H_17 cells (38). Due to the presence of microbiota in the intestine, the T_H_17 cell differentiation differs in comparison to sterile organs. One essential alteration is the activation of the transcription factor aryl hydrocarbon receptor (AHR) by ligands derived from food or intestinal microbiota (39, 40). AHR is highly expressed already in early stages of T_H_17 cell differentiation (41, 42). AHR expression is not essential for T_H_17 cell differentiation. However, it is nonetheless non-redundant for the secretion of IL-22 by T_H_17 cells, a cytokine vital for the anti-microbial properties of T_H_17 cells (42–44).

IL-23 is an important cytokine for T_H_17 cell biology. However, the IL-23 receptor is absent on naïve T cells. Accordingly, research led to the discovery that IL-23 is essential for the effector properties of T_H_17 cells rather than their induction (45, 46).

In the following sections, we want to outline the effects of T_H_17 cell-associated cytokines such as IL-17A, IL-22, and TNF-α on epithelial cells during IBD and carcinogenesis.

## IL-17A During Inflammation and Carcinogenesis

IL-17A is a member of the IL-17 family consisting of IL-17A, IL-17-B, IL-17-C, IL-17-D, IL-17E, and IL-17F (47). Both IL-17A and IL-17F signal through the IL-17RA–IL-17RC complex and activate the NF-κB and MAPK pathways (48). IL-17A is produced mainly by T_H_17 cells although production by many other cell types including CD8^+^ T cells, γδ T cells, NK cells, NKT cells, and innate lymphoid cells (ILCs) has been described. Initial studies have shown increased *IL17A* mRNA expression and increased numbers of T_H_17 cells in the inflamed tissue of IBD patients compared to healthy mucosa (18, 49, 50). Furthermore, the amount of IL-17A producing PBMCs correlates with disease severity in patients with UC (51). These results imply a pathogenic role of those cells in the intestine in IBD. IL-17A induces the recruitment and activation of granulocytes and locally promotes the production of other pro-inflammatory cytokines such as TNF-α, IL-6, and IL-1β (52, 53). In line with these findings, a blockade of IL-23 and IL-21 in murine models of colitis results in decreased numbers of T_H_17 cells and in a favorable disease outcome (54, 55). Surprisingly however, blockade or genetic deletion of IL-17A resulted in aggravated disease severity in the DSS-induced colitis, a mouse model of IBD (55, 56). Interestingly and in contrast with the data obtained using IL-17A knock-out mice, IL-17F knock-out mice show a less severe DSS-induced colitis (57). As IL-17F binds to the IL-17RA-IL-17RC complex with lower affinity than IL-17A, the different activation strength of the receptor complex might explain those opposing results. These findings do emphasize the need for strict distinction between the functions of T_H_17 cells and one of their signature cytokines, IL-17A. The protective function of IL-17A in mouse IBD models can be explained by the effect of this cytokine on enterocytes. Similar to IL-22, IL-17A induces their proliferation and tight-barrier formation and therefore promotes the integrity of the epithelial barrier (55, 58). The other side of the coin of those regenerative and physiological effects is the potential of IL-17A to promote carcinogenesis in the colon. Following a barrier defect, bacterial translocation and IL-23 production by innate immune cells, such as DCs and macrophages (Mφ), induce high levels of IL-17A which in turn can favor intestinal tumorigenesis (59). Importantly, an enterocyte-specific knock-out of the IL-17RA decreased tumor formation in mice, suggesting a direct tumorigenic function of IL-17A (60). Moreover, increased frequencies of IL-17A producing cells were found in human CRC and high *IL17A* mRNA expression correlates with a poor prognosis in these patients (60–62). Although these results do indicate a potential therapeutic benefit of targeting IL-17A, the heterogeneity of T_H_17 cells makes it difficult to conclude their exact function in human CRC and further research is needed before initiating clinical trials.

## IL-22 During Inflammation and Carcinogenesis

**Figure 1:**
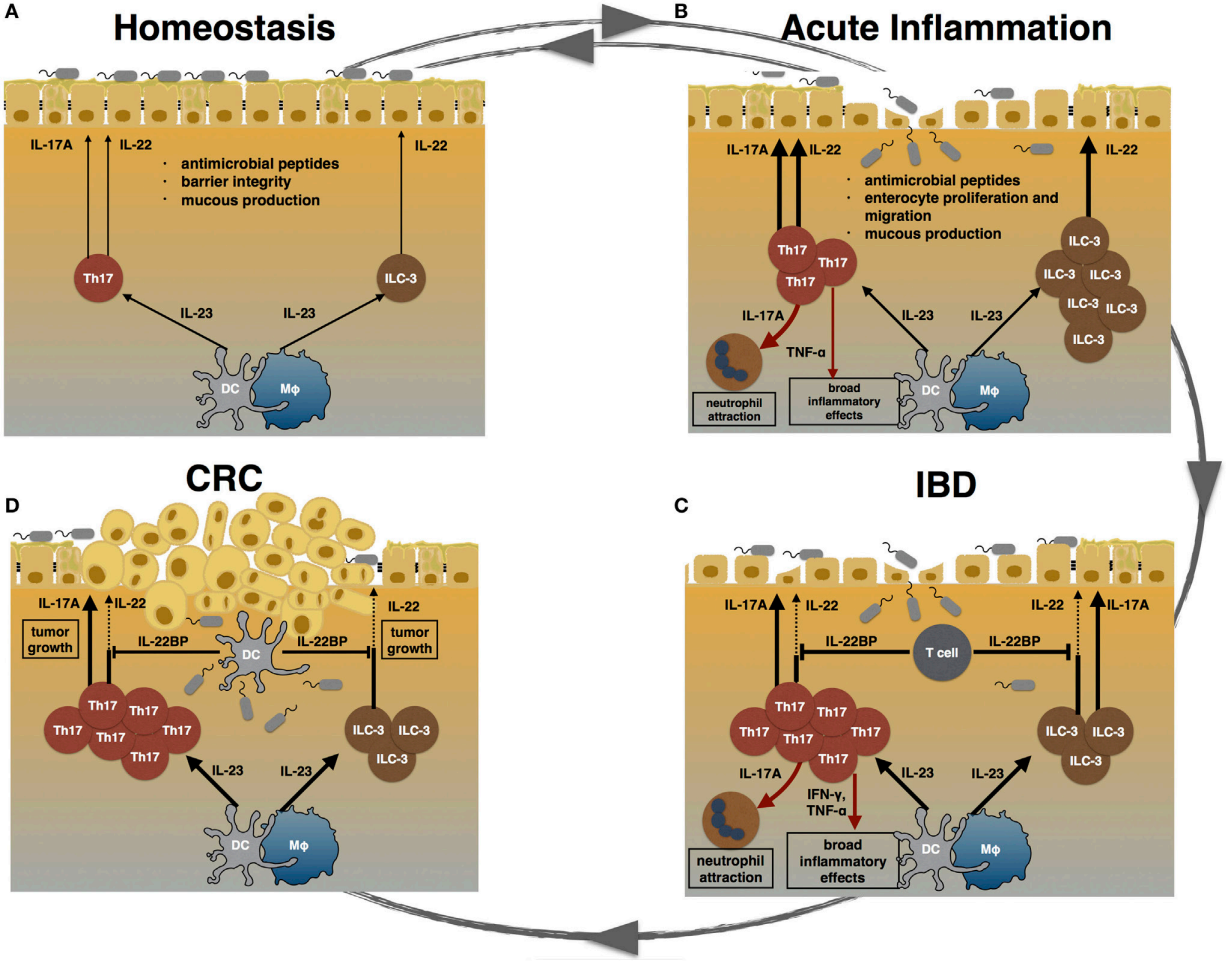
TH17 cells during homeostasis, inflammation, and carcinogenesis. **(A)** During homeostasis, TH17 cells and ILC3 are induced by certain species of the commensal microbiota. IL-17A and IL-22 promote epithelial barrier integrity, mucus production, and the release of anti-microbial peptides. **(B)** Acute inflammation can be caused by pathogenic bacteria. Invading pathogens induce the expansion of TH17 cells and ILC3. TH17 cell-associated cytokines attract neutrophils and trigger a pro-inflammatory response in order to clear the invading agent. Furthermore, IL-17A and IL-22 promote enterocyte proliferation and migration, thereby promoting mucosal healing. After clearance of the pathogen, the intestinal immune system returns to homeostasis. **(C)** Failure to terminate an intestinal immune response can lead to chronic inflammation. In inflammatory bowel disease (IBD), highly pathogenic TH17 cells expand and secret pro-inflammatory cytokines such as IL-17A, TNF-α, and IFN-γ. Especially, TNF-α and IFN-γ cause a broad inflammatory response. The regenerative effects of IL-22 are counter regulated by high levels of T cell-derived IL-22 binding protein (IL-22BP) in IBD patients. **(D)** Chronically elevated levels of IL-17A and IL-22 can promote carcinogenesis. Hereby, IL-22 can be controlled by DC-derived IL-22BP. However, whether this mechanism fails in human colorectal cancer (CRC) is currently unknown.

IL-22 is a member of the IL-10 family that has gained considerable attention in the last years due to its role in linking inflammation and regenerative processes. Although originally described as a T_H_1 cytokine (63), it can be produced by a variety of immune cells including CD4^+^ (T_H_1, T_H_17, T_H_22), CD8^+^ T cells, γδ T cells, NK cells, NKT cells, and group 3 ILCs. Innate lymphoid cells represent a newly described cell type which belongs to the lymphoid lineage but is characterized by the absence of antigen-specific T- or B-cell receptors. Group 3 ILCs represent a subgroup of innate lymphoid cells that are defined by the expression of the master transcriptional factor RORγt and the capacity to produce IL-17A and IL-22. They are therefore widely regarded as the innate counterpart of Th17 cells. Based on the expression of NKp46 (NCR1), group 3 ILCs can be further subdivided into ILC3s and lymphoid-tissue inducer cells. IL-22 signals through a heterodimeric receptor consisting of the ubiquitously expressed IL10R2 and the more specifically expressed IL22R1 (64, 65). Upon binding of IL22, the IL-22–IL-22R1–IL-10R2 complex signals mainly through STAT3 and the AKT-MAPK pathway, although activation of STAT1 and STAT5 has also been shown in certain cells (66, 67). The need for tight control of IL-22 activity is emphasized by the existence of an endogenous antagonist, called IL-22 binding protein (IL-22BP), which binds IL-22 and prevents binding to the membrane bound IL-22 receptor. In the intestine, IL-22 signaling elicits multiple responses that aim to maintain the integrity of the mucosal barrier, trigger antimicrobial responses, and promote wound healing (68, 69). In mice, levels of IL-22 increase upon chemically induced tissue damage and are crucial for tissue regeneration. Interestingly, levels of IL-22BP decrease in parallel, further increasing the activity of IL-22 (70). Similar results have been described in patients with acute diverticulitis (71). Surprisingly however, IL-22BP levels were recently shown to be elevated in patients with IBD and thus limit the tissue-healing effects of IL-22, suggesting that a disturbed IL-22–IL-22BP axis might be one of the mechanisms contributing to the chronification of inflammation (Figure 1) (71, 72). Although usually regarded as a tissue-protective cytokine, pro-inflammatory effects of IL-22 in the intestine have also been described. For instance, NCR-ILC-3, which coproduce IL-17A and IL-22, have been shown to be pathogenic in mouse models of IBD in a microbiota-dependent fashion and are increased in patients with IBD (73, 74). On the other hand, NCR-ILC-3 decreased in these patients and depletion of IL-22 producing ILC-3 makes mice more susceptible to *C. rodentium* infection (75–77). Similarly, T cell-derived IL-22 was shown to be both pathogenic and protective in murine models of IBD (78, 79). The distinct functions of ILC and T cell-derived IL-22 in IBD are still not completely understood. It can be hypothesized that ILC-derived IL-22 is crucial in the initial phases of inflammatory responses, whereas in chronically inflamed tissue, T cell become the major producers as suggested by Basu et al. (80).

As mucosal healing is one of the major aims in the therapy of IBD, IL-22 is widely regarded as a potentially beneficial cytokine in those diseases. On the other hand, a relationship between regenerative agents and tumorigenesis has been known for a long time and patients with IBD are known to have an increased risk for the development of CRC. Indeed, prolonged or excessive IL-22 signaling promotes tumorigenesis in the intestine of both mice and humans. In murine models of colon cancer, knock-out of the IL-22BP resulted in accelerated tumor growth, which was IL-22 dependent (70). In line with these results, levels of IL-22 are increased in patients with CRC and IL-22 can directly promote growth of cancer cell lines *in vitro* (81). Although ILCs have been shown to promote tumor growth in mice through IL-22, data in humans indicate CCR6^+^ T_H_17 cells to be the major source (81, 82). Further research is required to understand the distinct effects of ILC vs. T cell derived IL-22 in human carcinogenesis.

## TNF-α During Inflammation and Carcinogenesis

The cytokine TNF-α has been linked to inflammatory responses for a long time. TNF-α can be produced by a multitude of immune cells such as macrophages, CD4^+^ lymphocytes, NK cells, neutrophils, mast cells, and eosinophils. A soluble form and transmembrane bound form of TNF-α exist (83). Both the soluble and the membrane-bound TNF-α can interact with two TNF receptors, TNF-R1 (TNFRSF1A) and TNF-R2 (TNFRSF1B), expressed on IEC (84). TNF-α signaling leads to the release of other pro-inflammatory molecules and can provoke both pro- and anti-apoptotic signals in the IEC (85). An involvement of TNF-α in the pathology of IBD was first assumed after assessing TNF-α levels in serum of children suffering from IBD (86). Further studies revealed elevated levels of TNF-α in stool and mucosal tissue also (87, 88). Based on the knowledge of the pro-inflammatory properties and these observations, TNF-α became an interesting target for new therapeutic approaches. The development of anti-TNF-α antibodies and their application in humans is one of the best examples of how the concept, “from bench to bedside” can be successfully employed. Already in 1995 Dullemen et al. reported the successful use of a monoclonal antibody cA2 (infliximab) in CD patients (89). Since then, anti-TNF-α therapy has greatly improved the management of IBD. Furthermore, it has been demonstrated that TNF-α can promote colitis-associated CRC, a long-term consequence of IBD. In a mouse model of colitis-induced carcinogenesis, the blockade of TNF-α led to reduced mucosal injury and in turn to decreased tumor formation (90). Accordingly, elevated expression of TNF-α in tumors of CRC patients is associated with advanced cancer stages in humans (91). However, the treatment with anti-TNF antibodies causes high costs to health care systems and can cause some severe side effects such as opportunistic infections (92). Finally, around 10–30% of patients with IBD do not respond to anti-TNF-α treatment and 20–40% of patients lose response over time (93). Moreover, a prognostic factor that can predict the response to this therapy is missing.

## New Therapeutic Strategies and Patient Management

T_H_17 cells are highly enriched in IBD, and several genetic risk loci being associated with IBD are linked to T_H_17 cells. Thus, several drugs were developed with the intention to manipulate T_H_17 cell development or function in patients with IBD. First clinical trials using monoclonal antibodies targeting cytokines related to T_H_17 cells, such as IL-17A, show similar results to those obtained in murine IBD models and highlight the need to make a clear distinction between the biological functions of IL-17A and T_H_17 cells in general. As described above, despite the potential pro-inflammatory properties of T_H_17 cells, their signature cytokine IL-17A was shown to also have beneficial effects. IL-17A can promote enterocyte proliferation, tight-barrier formation and epithelial barrier integrity in the intestine (54, 55). A clinical trial with secukinumab, an anti-IL-17A antibody, further highlighted the importance of IL-17A for mucosal homeostasis. Although being highly effective in psoriasis, blockade of IL-17A resulted in aggravated disease course in IBD patients (94–96). In contrast to blockade of IL-17A, antibodies against IL-23 were effective in preventing colitis in mouse models (55). Similarly, ustekinumab proved to be effective in patients with CD (97). Ustekinumab targets the p40 subunit of IL-12, which is part of IL-12 and IL-23. Therefore, it affects both T_H_1 (together with ILC-1) and T_H_17 (together with ILC-3) lineages. Interestingly, a first trial involving the blockade of the p19 subunit of IL-23 (and therefore not affecting IL-12 signaling) using risankizumab also delivered promising results in patients with moderate-to-severe CD and might represent a future therapy in IBD (98). In contrast to the above-mentioned approaches, anti-TNF therapy is already well established in the therapy of both CD and UC. Surprisingly, we have recently shown that the effectiveness of this therapy is to some extent dependent on the IL-22–IL-22BP axis in both mice and humans (71). Direct therapeutic intervention into the IL-22–IL-22R1–IL-22BP system might therefore represent a novel strategy in patients with IBD. Importantly, as IL22R1 is expressed exclusively on non-hematopoietic cells, such a therapy would not lead to systemic immunosuppression, a common side-effect of most currently used medications. Nevertheless, the oncogenic properties of IL-22 could represent a major obstacle in the development of safe and effective drugs targeting the IL-22–IL-22R1–IL-22BP axis.

## Author Contributions

LB and JK wrote the manuscript and equally contributed to the manuscript; NG and SH supervised and revised the manuscript.

## Conflict of Interest Statement

The authors declare that the research was conducted in the absence of any commercial or financial relationships that could be construed as a potential conflict of interest.
